# Push-Dose Pressors During Peri-intubation Hypotension in the Emergency Department: A Case Series

**DOI:** 10.5811/cpcem.2021.4.51161

**Published:** 2021-10-28

**Authors:** Abdullah Bakhsh, Leena Alotaibi

**Affiliations:** * The King Abdulaziz University, Department of Emergency Medicine, Jeddah, Saudi Arabia; † The King Abdulaziz University, Faculty of Medicine, Jeddah, Saudi Arabia

**Keywords:** Push-dose pressors, peri-intubation hypotension, push-dose epinephrine, push-dose phenylephrine

## Abstract

**Introduction:**

Emergency physicians frequently encounter critically ill patients in circulatory shock requiring definitive airway procedures. Performing rapid sequence intubation in these patients without blood pressure correction has lethal complications. Questioning the efficacy and fearing side effects of push-dose pressors (PDP) has created an obstacle for their use in the emergency department (ED) setting. In this case series we describe the efficacy and side effects of PDP use during peri-intubation hypotension in the ED.

**Case series:**

We included 11 patients receiving PDPs in this case series. The mean increase in systolic blood pressure was 41.3%, in diastolic blood pressure 44.3%, and in mean arterial pressure 35.1%. No adverse events were documented in this case series.

**Conclusion:**

The use of push-dose pressors during peri-intubation hypotension may potentially improve hemodynamic status when used carefully in the ED.

## INTRODUCTION

Rapid sequence intubation (RSI) is the cornerstone of emergency airway management. Nonetheless, if not executed carefully the complications can be deadly.^1^ In one study, patients with systolic hypotension (<90 millimeters of mercury [mmHg]) during RSI were more likely to sustain post-intubation cardiac arrest. The multivariate analysis showed that systolic hypotension was independently associated with post-intubation cardiac arrest (3.7 [95% confidence interval (CI), 1.6–8.6]; *P* = 0.01).^2^,^3^ For many years, push-dose pressors (PDP) have been used in the operating room for rapid blood pressure correction.^4^,^5^ This practice, however, has yet to be translated into standard emergency medicine practice.^6^

The emergency department (ED) environment poses unique challenges in ensuring seamless and safe administration; treatment of unfamiliar patients, crowding, reliance on verbal orders, dispensing and administering medications without verification by a pharmacist, and understaffing.^7^ A major reason for discouraging the use of such medications is that they add additional patient safety risks in addition to the complex, multistep process of using bolus-dose pressors.^7^,^8^ Risks include the need for dose calculation, drug dilution, and incremental push-dose administration. These are all areas in which errors are common and potentially fatal.^9^,^10^ In this case series we assessed the effect of PDP on blood pressure during RSI (defined as blood pressure change of at least 20%). We additionally assessed for any side effects including tachyarrhythmias, local tissue injury, and errors.

## CASE SERIES

The use of PDP during peri-intubation hypotension was part of a quality improvement program within the ED. We implemented a RSI bundle; this included instructions for PDP mixing (Supplementary). These instructions were permanently attached to each intubation cart in the department, allowing immediate access. Additionally, an endotracheal intubation procedure note was completed for every patient requiring intubation by the emergency physician. We collected data retrospectively from intubation procedure notes. The notes included the following: patient information; diagnosis; indication for intubation; pre- and post-intubation vital signs; pre- and post-intubation drugs (with dose and time); number of intubation attempts; device used; operator level; and complications.

Push-dose pressors are prepared (following mixing instructions) and administered via large bore (14–18 gauge) peripheral intravenous (IV) access over one minute by the primary nurse. Vital signs were documented in the note at five minutes before and after the procedure. Adverse effects including extravasation and dysrhythmias were monitored for 30 minutes of PDP administration; standard procedure for nursing staff is to monitor for these adverse effects and document in the electronic health record if they do occur. Study investigators raised awareness of the new RSI bundle and PDP mixing instructions via departmental educational activities, bedside clinical demonstration, didactic lectures, and procedure note review. The bundle was approved by the department’s chair and clinical practice committee.

We included patients undergoing the following: 1) RSI in the ED; or 2) receiving PDPs for a systolic blood pressure less than or equal to 100 mm Hg and/or diastolic blood pressure less than or equal to 60 mm Hg. During the one-year study period (January 2019–January 2020), a total of 86 patients underwent RSI by emergency physicians. Of those, only 11 patients received PDP for hypotension (defined as blood pressure less than or equal to 100/60 mm Hg within five minutes prior to the procedure). Table 1 describes the general characteristics of these patients. Table 2 describes the change in hemodynamic parameters in relation to PDP.

**CPC-EM Capsule**

What do we already know about this clinical entity?

*Push-dose pressor (PDP) is common practice for rapid hemodynamic correction in the operating room. However, there is scant research examining the use of PDP in the emergency department.*

What makes this presentation of disease reportable?

*Understanding the effects of PDPs during*

*peri-intubation hypotension will aid emergency physicians in clinical practice.*

What is the major learning point?

*Consider careful use of PDP for peri-intubation hypotension in the ED.*

How might this improve emergency medicine practice?

*Rapid correction of hemodynamic parameters prior to rapid sequence intubation, is crucial to lower deadly complications such as peri-intubation cardiac arrest.*

### Efficacy

We assessed the efficacy based on pre- and post-PDP vital signs in 11 patients (Table 3). The overall mean change in systolic blood pressure was 33.5 mm Hg (41.3% increase from baseline), the change in diastolic blood pressure was 21.4 mm Hg (44.3% increase from baseline), and in mean arterial pressure the change was 21.5 mm Hg (35.1% increase from baseline).

### Safety

It is standard procedure to document any side effects from medication administration given intravenously. This is especially true for high-risk medications such as peripheral vasopressors. No events of extravasation or local tissue injury were documented in patients receiving PDP via peripheral IV route. Additionally, no documented cardiac dysrhythmias were found.

## DISCUSSION

Significant data exist on the utilization and benefit of phenylephrine for hypotension induced by spinal anesthesia and neurologic emergencies.^11^,^12^ However, only scant scientific evidence examines the use of PDP for peri-intubation hypotension in the ED.^6^ This case series describes a one-year cohort of patients receiving PDP for peri-intubation hypotension. We found a mean increase from baseline in all hemodynamic parameters with the use of PDP: systolic blood pressure increased by 41.3%; diastolic blood pressure increased by 44.3%; and mean arterial pressure increased by 35.1%. Rotando et al^13^ evaluated PDP practice patterns and sought to determine the efficacy in hospitalized hypotensive patients outside of the operating room. Results show a mean increase in systolic blood pressure of 32.5% and a mean increase in diastolic blood pressure of 27.2%. Our series shows a larger mean increase in hemodynamic parameters. This is explained by the different agents used in both studies. The most frequently used PDP in this series is epinephrine (10 out of 11 patients). Previous reports, including those by Rotando et al, mostly used phenylephrine and ephedrine.^6^,^13^

## LIMITATIONS

First, this is a small sample size without comparison and therefore results are not generalizable. Second, the most commonly used PDP was epinephrine in 10 patients, while phenylephrine was only used in 1 patient. Third, this case series lacks data on patients requiring continuous vasopressor infusion after PDP. Fourth, dysrhythmias and extravasation was only monitored for 30-minutes post administration. While these PDPs are short-acting it is unclear if side effects may develop after the 3-minute time point.

## CONCLUSION

Based on this small case series we conclude that the use of push-dose pressors causes a significant increase in systolic, diastolic, and mean arterial blood pressure (defined as >20% change). Therefore, if implemented with strict monitoring guidelines and appropriate education, we do not anticipate significant adverse events.

## Supplementary Information



## Figures and Tables

**Table 1:** General characteristics of patients undergoing rapid sequence intubation.

Patient No.	Age/Gender	Indication for intubation	Medications	

			Induction	Paralytic
1	57 F	Anticipated clinical course	100 mg ketamine	50 mg rocuronium
2	59 F	Hypoxic respiratory failure	20 mg etomidate	40 mg rocuronium
3	78 M	Anticipated clinical course	20 mg etomidate	100 mg succinylcholine
4	42 M	Anticipated clinical course	30 mg etomidate	100 mg succinylcholine
5	61 F	Hypoxic respiratory failure	20 mg etomidate	100 mg succinylcholine
6	62 M	Airway protection	20 mg etomidate	100 mg succinylcholine
7	63 M	Anticipated clinical course	20 mg etomidate	100 mg succinylcholine
8	66 F	Anticipated clinical course	20 mg etomidate	100 mg succinylcholine
9	60 M	Airway protection	20 mg etomidate	100 mg succinylcholine
10	51 M	Airway protection	20 mg etomidate	100 mg succinylcholine
11	71 F	Anticipated clinical course	20 mg etomidate	100 mg succinylcholine

*M*, male*; F,* female*; mg,* milligrams*.*

**Table 2:** Change in hemodynamic parameters in relation to push-dose pressors.

Patient no.	Pre-Push Dose Hemodynamics				PDP	Post-Push Dose Hemodynamics			
	
	SBP	DBP	MAP	HR		SBP	DBP	MAP	HR
1	81	51	51	81	10 mcg epinephrine	174	94	121	128
2	70	50	50	70	10 mcg epinephrine	84	62	69	146
3	90	62	71	95	20 mcg epinephrine	108	85	93	98
4	93	50	64	62	40 mcg epinephrine	104	55	71	62
5	76	35	49	106	10 mcg epinephrine	72	36	48	110
6	82	51	61	130	20 mcg epinephrine	106	68	81	114
7	83	46	58	63	200 mcg phenylephrine	120	57	78	84
8	87	52	64	52	10 mcg epinephrine	97	76	83	130
9	81	48	59	91	10 mcg epinephrine	153	76	102	87
10	65	40	48	120	10 mcg epinephrine	146	97	113	86
11	84	46	59	100	10 mcg epinephrine	96	60	72	89

*PDP*, push-dose pressor*; SBP,* systolic blood pressure (millimeters of mercury [mm Hg)]);* DBP,* diastolic blood pressure (mm Hg)*; MAP,* mean arterial pressure (mm Hg*); HR*, heart rate (beats per minute*); mcg,* micrograms*.*

**Table 3:** Overall mean hemodynamic parameters (before and after PDP*) with mean percent change.

Hemodynamic parameter	Blood pressure value and percent change with push-dose pressor
Pre-SBP	81.1 mm Hg
Post-SBP	114.5 mm Hg
Percent change	41.3%
Pre-DBP	48.3 mm Hg
Post-DBP	69.6 mm Hg
Percent change	44.3%
Pre-MAP	61.2 mm Hg
Post-MAP	82.6 mm Hg
Percent change	35.1%

*PDP, push-dose pressor; SBP,* systolic blood pressure;* DBP,* diastolic blood pressure;* MAP,* mean arterial pressure.

## References

[1] Holler JG, Bech CN, Henriksen DP (2015). Nontraumatic hypotension and shock in the emergency department and the prehospital setting, prevalence, etiology, and mortality: a systematic review. PLoS One.

[2] Marchick MR, Kline JA, Jones AE (2009). The significance of non-sustained hypotension in emergency department patients with sepsis. Intensive Care Med.

[3] Jones AE, Yiannibas V, Johnson C (2006). Emergency department hypotension predicts sudden unexpected in-hospital mortality: a prospective cohort study. Chest.

[4] Siddik-Sayyid SM, Taha SK, Kanazi GE (2014). A randomized controlled trial of variable rate phenylephrine infusion with rescue phenylephrine boluses versus rescue boluses alone on physician interventions during spinal anesthesia for elective cesarean delivery. Anesth Analg.

[5] Heesen M, Stewart A, Fernando R (2015). Vasopressors for the treatment of maternal hypotension following spinal anaesthesia for elective caesarean section: past, present and future. Anaesthesia.

[6] Panchal AR, Satyanarayan A, Bahadir JD (2015). Efficacy of bolus-dose phenylephrine for peri-intubation hypotension. J Emerg Med.

[7] Weant KA, Bailey AM, Baker SN (2014). Strategies for reducing medication errors in the emergency department. Open Access Emerg Med.

[8] Holden D, Ramich J, Timm E (2018). Safety considerations and guideline-based safe use recommendations for “bolus-dose” vasopressors in the emergency department. Ann Emerg Med.

[9] Kanwar M, Irvin CB, Frank JJ (2010). Confusion about epinephrine dosing leading to iatrogenic overdose: a life-threatening problem with a potential solution [published correction appears in *Ann Emerg Med*. 2010 Jul;56(1):23. Dosage error in article text]. Ann Emerg Med.

[10] Campbell RL, Bellolio MF, Knutson BD (2015). Epinephrine in anaphylaxis: higher risk of cardiovascular complications and overdose after administration of intravenous bolus epinephrine compared with intramuscular epinephrine. J Allergy Clin Immunol Pract.

[11] Habib A (2012). A Review of the impact of phenylephrine administration on maternal hemodynamics and maternal and neonatal outcomes in women undergoing cesarean delivery under spinal anesthesia, anesthesia & analgesia. Anesth Analg.

[12] Lee A, Ngan Kee W, Gin T (2002). Quantitative, systematic review of randomized controlled trials of ephedrine versus phenylephrine for the management of hypotension during spinal anesthesia for cesarean delivery. Anesth Analg.

[13] Rotando A, Picard L, Delibert S (2019). Push dose pressors: experience in critically ill patients outside of the operating room. Am J Emerg Med.

