# Supplementary material for: Push-Dose Pressors During Peri-intubation Hypotension in the Emergency Department: A Case Series
Source: Clin Pract Cases Emerg Med. 2021 Oct 28;5(4):390–3. doi: 10.5811/cpcem.2021.4.51161 (PMC8610482; doi:10.5811/cpcem.2021.4.51161)
Supplement: Supplementary file 1 [file cpcem-5-390-s001.pdf]

PUSH-DOSE PRESSORS

PHENYLEPHRINE

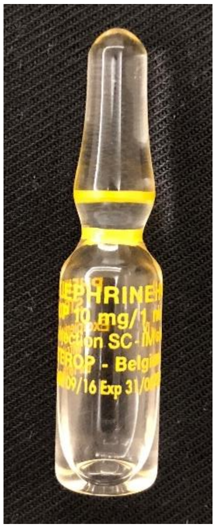

Mixing instructions

Draw 1 mL of Phenylephrine from amp (10 mg/mL) into a 3 mL syringe
Inject into a 100 mL bag of normal saline
Now you have 10 mg of phenylephrine in 100 mL (each mL contains 0.1 mg or 100 mcg of phenylephrine)
Draw up from bag into syringe for intravenous bolus dosing

Onset: 1-minute.

Duration 10-20 minutes.

Dose: 50-200 mcg IV (0.5-2 mL q 2-5 min).

PUSH-DOSE PRESSORS

EPINEPHRINE

0.1 mg/mL (1 :10,000)

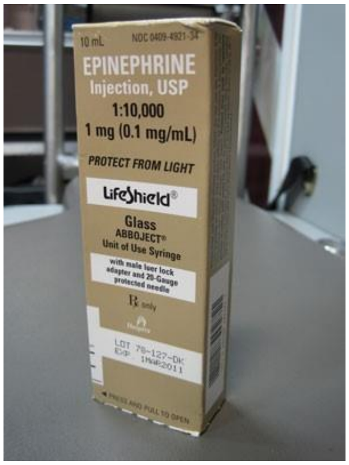

Mixing instructions

Draw 9 mL of normal saline into a 10 mL syringe
Into this syringe, draw 1 mL of epinephrine (0.1 mg/mL)
Preparation: 10 mL of Epinephrine (10 mcg/mL)
Ready to use for intravenous bolus dosing

Onset: 1-minute.

Duration: 5-10 minutes.

Dose: 5-20 mcg IV (0.5-2 mL q 2-5 min).

PUSH-DOSE PRESSORS

EPINEPHRINE
1 mg/mL (1 :1000)

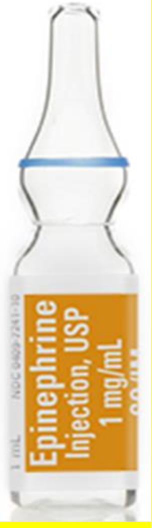

Mixing instructions

Draw 1 mL of epinephrine ampule (1 mg/mL, or 1:1000) into a 3 mL syringe
Inject into 100 mL bag of normal saline (now you have ~ 1 mg of epinephrine in 100 mL of normal saline)
Draw 1 mL from the bag into a syringe (each 1 mL in the syringe contains 0.01 mg or 10 mcg of epinephrine)
Ready to use for intravenous bolus dosing (bag is stable for 24 hours)

Onset: 1-minute.

Duration: 5-10 minutes.

Dose: 5-20 mcg IV (0.5-2 mL q 2-5 min).
